# Electrochemical Membrane Reactors for Sustainable Chlorine Recycling

**DOI:** 10.3390/membranes2030510

**Published:** 2012-07-30

**Authors:** Tanja Vidakovic-Koch, Isai Gonzalez Martinez, Rafael Kuwertz, Ulrich Kunz, Thomas Turek, Kai Sundmacher

**Affiliations:** 1 Max Planck Institute for Dynamics of Complex Technical Systems, Process Systems Engineering, Sandtorstrasse 1, Magdeburg 39106, Germany; Email: sundmacher@mpi-magdeburg.mpg.de; 2 Chair for Process Systems Engineering, Otto von Guericke University, Universitätsplatz 2, Magdeburg 39106, Germany; Email: gonzalez@mpi-magdeburg.mpg.de; 3 Institute of Chemical Process Engineering, Clausthal University of Technology, Leibnizstr. 17, Clausthal-Zellerfeld 38678, Germany; Email:kuwertz@icvt.tu-clausthal.de (R.K.); kunz@icvt.tu-clausthal.de (U.K.); turek@icvt.tu-clausthal.de (T.T.)

**Keywords:** polymer electrolyte membrane, Nafion, HCl electrolysis, fuel cell, hydrochloric acid, hydrogen chloride, chlorine, recycling, catalyst layer, three phase boundary, crossover

## Abstract

Polymer electrolyte membranes have found broad application in a number of processes, being fuel cells, due to energy concerns, the main focus of the scientific community worldwide. Relatively little attention has been paid to the use of these materials in electrochemical production and separation processes. In this review, we put emphasis upon the application of Nafion membranes in electrochemical membrane reactors for chlorine recycling. The performance of such electrochemical reactors can be influenced by a number of factors including the properties of the membrane, which play an important role in reactor optimization. This review discusses the role of Nafion as a membrane, as well as its importance in the catalyst layer for the formation of the so-called three-phase boundary. The influence of an equilibrated medium on the Nafion proton conductivity and Cl^−^ crossover, as well as the influence of the catalyst ink dispersion medium on the Nafion/catalyst self-assembly and its importance for the formation of an ionic conducting network in the catalyst layer are summarized.

## 1. Introduction

Membranes are omnipresent in nature. For example, cell membranes control fluxes in and out of the cell acting as cell gate-keepers. To facilitate their gate-keeping function, cell membranes are equipped with special functionalities, which may provide for selective ion channeling or proton pumping through the membrane. Membranes, as a naturally inspired solution, have also found applications in technical processes. However, technical membranes are not as sophisticated as natural membranes. The challenge remains to develop as efficient technical membranes as those provided by nature.

Like in biological systems, the primary function of membranes in electrochemical processes is separation. The second equally important function is the conduction of ions. These two main functions define the most important properties of membranes under electrochemical conditions *i.e.*, high separation efficiency and high ionic conductivity. In addition, membranes should possess high mechanical and chemical stability to withstand harsh technical conditions.

The focus of this contribution is on polymer electrolyte membranes, which have found applications in different low-temperature processes so far. Among these materials, perfluorosulfonic acid membranes such as Nafion were especially the subject of intensive research in the past. Nafion membranes were developed by the DuPont Company more than 40 years ago for application in electrochemical membrane separation processes [1,2,3]. Patent literature not only describes the preparation of these membranes, but also describes the preparation of Nafion solutions which were originally used for membrane casting or to repair membrane defects [4]. Today, Nafion solutions are used for gas diffusion electrode preparation in order to improve the three-phase boundary in the catalyst layer. Although the earliest research and development of Nafion was concerted on its application in electrochemical membrane reactors for large-scale industrial chlorine production, recent efforts aim at its application as a proton conducting membrane in fuel cells. However, the employment of Nafion in electrochemical separation processes recently has become of renewed interest. We further address recent electrochemical membrane processes for chlorine production. 

Chlorine is a platform chemical with about 15 000 chlorine compounds being used commercially [5]. It takes part in the production of a wide range of industrial and consumer products like plastics, solvents for dry cleaning and metal degreasing, textiles, agrochemicals and pharmaceuticals, insecticides, dyestuffs, household cleaning products, *etc.* [6]. Chlorine is industrially mainly obtained through the electrolysis of brine with chlorine, hydrogen and caustic soda as main products [7]. This process is nowadays conducted in electrochemical membrane cells with Nafion as a membrane and it is industrially established. Still the process is energetically very demanding [7], and new process alternatives are under research [8]. One alternative considers the use of hydrochloric acid (HCl) as the chlorine source. This idea is very attractive, as HCl is a by-product in some industrial processes (e.g., in the production of polyurethanes and polycarbonates), which makes recycling important from the point of view of sustainability. 

**Figure 1:**
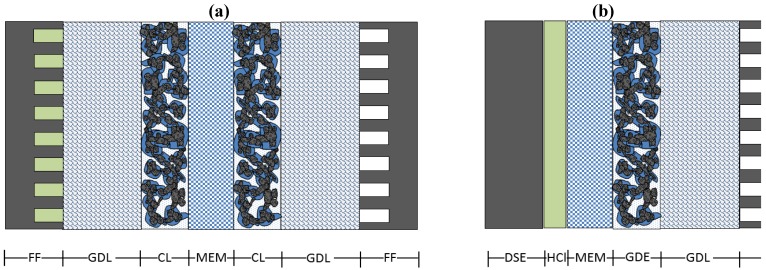
Schematic presentations of different reactor types used for HCl electrolysis: (**a**) dimensionally stable electrodes–gas diffusion electrodes (DSE–GDE) and (**b**) gas diffusion electrodes–gas diffusion electrodes (GDE–GDE) configuration.

Electrochemical chlorine recycling takes place in electrochemical reactors of similar construction as fuel cells (Figure 1) with the employment of gas diffusion electrodes (GDE). For this reason, some typical fuel cell problems, such as the optimization of GDE and the water management in the membrane, will also be of importance here, and, in some cases reference will be made to “fuel cell” literature. New issues, which arise, are mainly related to the “new chemistry” of these processes. In the case of chlorine production, for example, the crossover of chlorine anions through the membrane might affect catalysts. Below, we address some challenges in the development of the above-discussed aspects, such as proton conductivity, crossover, and GDE optimization.

## 2. Chlorine Recycling in an Electrochemical Membrane Reactor

HCl can be used in a gas or liquid (dissolved in water) state. The net reaction and half reactions can be expressed as follows:

**Overall** 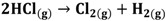 (**1**)

Cathode 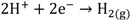 (1-1)

Anode 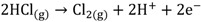 (1-2)

**Overall** 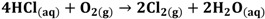 (**2**)

Cathode 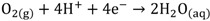 (2-1)

Anode 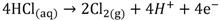 (2-2)

**Overall** 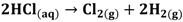 (**3**)

Cathode 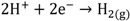 (3-1)

Anode 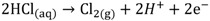 (3-2)

Equations 1 and 3 depict the direct conversion of hydrogen chloride (HC1_(g)_) and hydrochloric acid (HC1_(1)_), respectively, into its elements chlorine and hydrogen. Equation 2 follows the stoichiometry of the Deacon process, which was introduced in 1868 by Henry Deacon for the chemical chlorine production by the heterogeneously catalyzed oxidation of anhydrous HCl. Due to the high requirements on catalysts, the high temperatures, and the high demands for product purification, the Deacon process was soon outperformed by electrochemical processes. Newly, this idea has been successfully revisited due to the advances in ruthenium-based catalysts. For the recent progress in the field of heterogeneous chemical chlorine recycling, the reader may refer to the works of Perez-Ramirez *et al.* and Motupally *et al.* [9,10]. 

**Figure 2:**
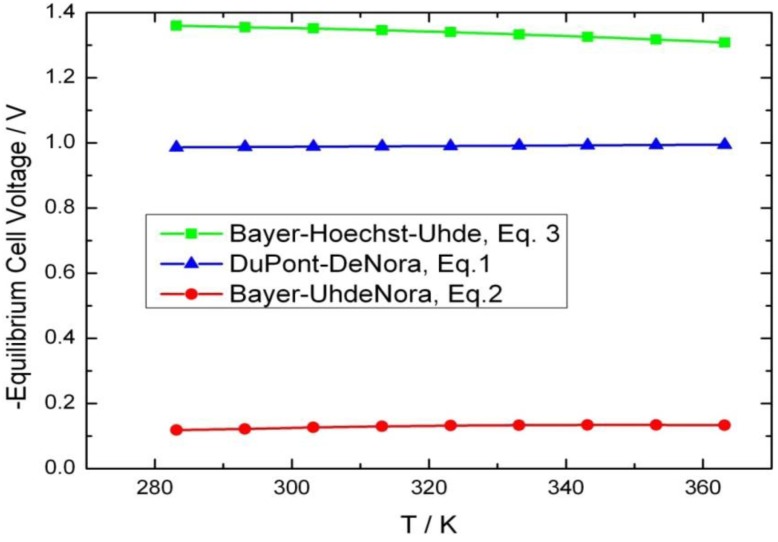
Influence of temperature on equilibrium cell voltage for different HCl electrolysis processes.

However, even with the present advances in heterogeneous chemical catalysis, electrochemical processes outperform chemical HCl oxidation in terms of the investment costs per ton of chlorine [9] and the possibility of module-based (decentralized) operation. In addition, the required process temperature is much lower in the electrochemical route (*ca.* 343 K compared to 453–773 K for the chemical process [9]), since the activation energy is supplied in form of electricity. The energy demand of an electrochemical process is determined by its thermodynamics, kinetics, and Ohmic losses. Equilibrium cell voltages at different temperatures, calculated based upon the thermodynamic data for the processes listed above (commercially known as the DuPont-DeNora (Equation 1), Bayer-Uhdenora (Equation 2), and Bayer-Hoechst-Uhde (Equation 3) process), are shown in Figure 2. 

As can be seen in Figure 2, the temperature effect on equilibrium cell voltage is negligible (0.1–0.6 mV K^−1^), and the Bayer-UhdeNora process utilizing an oxygen-consuming cathode is thermodynamically most favorable. Nevertheless, the kinetics of the oxygen reduction reaction (ORR) is more sluggish than the hydrogen reduction reaction (HRR), yielding the process rentable only at low current densities (between 300–400 mA cm^−2^ [10]). Thus, the electrolyzer performance is limited. 

Nafion membranes are employed in the DuPont-DeNora and Bayer-UhdeNora processes, while the Bayer-Hoechst-Uhde industrially still uses PVC-based diaphragms due to lower costs [11]. The electrochemical reactions 1-1, 1-2, and 2-1 take place on gas diffusion electrodes (GDE), while reaction 2-2 takes place on dimensionally stable electrodes (DSE). GDE are composed of a gas diffusion layer (GDL) and a catalyst layer (CL). The CL contains a catalyst (e.g., Pt), a solid electrolyte, and, in some cases, a supporting material (usually carbon black). In addition, flow fields (FF) are required to distribute reactants along the GDL, to provide physical support to the GDE, as well as to conduct electrons from the electrochemical reactions. DSE are composed of a bulk metal phase covered by a thin catalyst layer (usually Ti covered with ruthenium or iridium oxides [11]) and are typically placed 1 mm away from the membrane to allow for a reactant flow. Electrochemical reactors for chlorine recycling can be found in GDE–GDE or DSE–GDE configurations. The former one is able to handle gaseous or liquid HCl, the latter one only liquid. Both reactor types are schematically presented in Figure 1. 

The Nafion membrane in the above-shown reactor setups has the role of a separator and a solid electrolyte (as it conducts ions, ideally only protons). To extend a reaction zone, Nafion is also added to the catalyst layer and provides for a proton-conducting network. We address hereby both roles of Nafion as:

(a) A membrane, pointing out the following properties: the proton transport *i.e.*, the influence of an equilibrated medium, the Cl^−^ crossover and the mechanical stability of the membrane;
(b) A proton-conducting network in the catalyst layer, pointing out the influence of a dispersion medium on the self-assembly mechanism of the Nafion on the carbon supported catalysts, its relevance for the formation of an ion-conducting network in the catalyst layer, possibilities for its visualization by electrochemical and other methods and the role of Nafion as an ion-conducting medium.

### 2.1. Nafion as a Membrane

Nafion is a porous sulfonated tetrafluoroethylene-based polymer, which can be considered ideal for halogen electrolysis due to its chemical inertness in a highly corrosive atmosphere and its high cation permeability. In particular, Nafion 115, 117, 324, and 417 membranes have been employed for HCl electrolysis [12,13]. An important property of Nafion membranes is their proton conductivity. Nafion conducts protons but only in the presence of water. After extensive discussion regarding the proton transport mechanism within Nafion (refer to [14], for example), it has been accepted that the proton transport through the membrane fully depends upon the ionomer side chain motions at low water contents, while a proton hopping mechanism (so called “Grotthus mechanism”) is responsible for proton transport at high water contents. 

**Figure 3:**
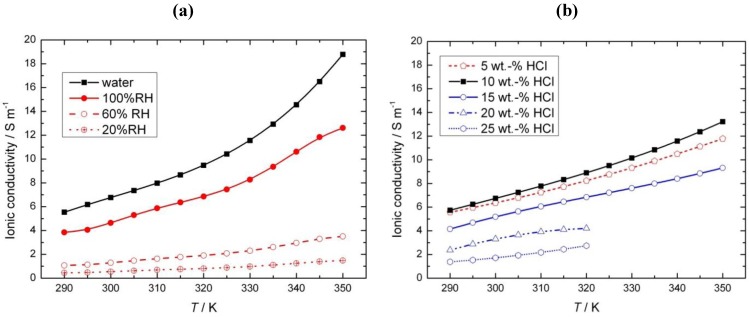
Influence of equilibrated medium on Nafion conductivity in the presence of (**a**) liquid water and vapor water at different relative humidities (RH) and (**b**) hydrochloric acid at different concentrations. Based on [14,15].

Nafion proton conductivity depends upon the type of equilibrated medium, e.g., liquid, vapor water or acid, as shown in Figure 3. The conductivity of the membrane equilibrated with water is usually employed in the literature as a reference value, and it ranges from 6 S cm^−1^ at room temperature to 19 S cm^−1^ at 80 °C. In the case of Nafion equilibrated with water vapor, slightly lower values that depend upon the relative humidity have been observed (Figure 3a). For HCl electrolysis, the data regarding the Nafion that is equilibrated with hydrochloric acid is especially interesting. As can be seen in Figure 3b, the Nafion conductivity reaches a maximum at about 10 wt % HCl, followed by a steep decrease. Similar to Nafion, hydrochloric acid shows a maximum in conductivity; however, this maximum is at around 20 wt % HCl [15]. In general, in the case of strong electrolytes, the appearance of a maximum can be explained by the formation of ion pairs at higher acid concentrations. In the case of the observed dependence of Nafion conductivity on the acid concentration, no satisfactory explanation has been offered so far. Nafion conductivity is approximately 10 times lower than is the case for liquid acid [15], and furthermore, according to the presented data, the conductivity of Nafion equilibrated with acid is lower (especially at higher acid concentrations) than that of Nafion equilibrated with water. 

**Figure 4:**
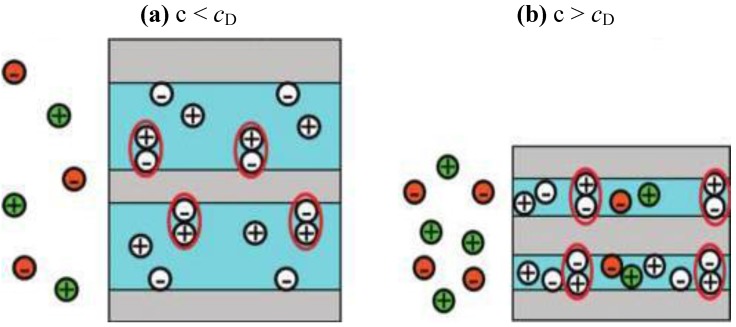
Schematic representation of the influence of an equilibrated medium concentration on the polymer electrolyte membrane structure (**a**) concentrations lower and (**b**) greater than the Donnan concentration *c_D_*. Reprinted with permission from reference [16].

The concentration of protons in the Nafion membrane is very high, namely 3.2 M [16], and for this reason, Nafion is considered to be a super-acid [17]. Still, to enable conductivity, these protons have to be present in a dissociated form. In general, it is believed that the proton transport in Nafion is enabled by the dissociation of all protons in the membrane. However, it has recently been reported [16] that only between 2% and 28% (depending on the method of determination) of all protons are dissociated in the case of the Nafion membrane equilibrated with water. In the same publication, the uptake of a hydrochloric acid solution by a Nafion 117 membrane was determined experimentally. At low acid concentrations (up to 0.03 M), the acid solution uptake was similar to that of pure water. The authors hypothesized that there is no driving force for ions from the solution to enter the membrane when the chemical potential of the ions in the solution is lower than that of the dissociated ions in the membrane, as depicted schematically in Figure 4a. At higher ion concentrations in the solution, their chemical potential will exceed that of the dissociated ions within the membrane, and the solution will enter the membrane (Figure 4b). The entering of ions into the membrane starts at a solution concentration denoted as the Donnan concentration (*c*_D_). At concentrations higher than the *c*_D_, the acid solution uptake starts to decrease, indicating the de-swelling of the membrane. The authors hypothesized that the presence of additional ions in the membrane would result in an increased screening of the charges inside the membrane and a shrinking of the hydrophilic channels. This was also confirmed experimentally by Small-Angle X-ray Scattering (SAXS) measurements and is shown schematically in Figure 4b. The mechanism of acid uptake suggested by these authors might qualitatively explain the observed experimental dependence of Nafion conductivity on the acid concentration (Figure 3b). At low acid concentrations, the conductivity should be close to the conductivity of water equilibrated Nafion (which corresponds to some extent to experimental observations) followed by a steep decrease of conductivity at higher concentrations (which has also been observed experimentally [Figure 3c]).

The importance of the above discussion for the behavior of Nafion under technical conditions is twofold. Firstly, Nafion conductivity will depend on the type of solution (and its concentration) in contact. Secondly, at higher acid concentrations, not only Nafion conductivity will decrease, but also the level of HCl crossover might increase, which might further influence the kinetics of a counterpart reaction. Furthermore, the application of high HCl concentrations in industrial electrolysis (18–20 wt % HCl solutions in e.g., the Bayer-UhdeNora process) implies significant Ohmic losses at technical current densities (300 to 400 mA cm^−2^) due to an almost 50% reduction in the Nafion conductivity at these concentrations. On the other hand, higher HCl concentrations at technical current densities suppress parasite O_2_ evolution on the anode side and increase the product selectivity.

Another parameter affecting the ionic conductivity of the membrane is the membrane pre-treatment. Yeo and McBreen [15] found that heating a Nafion membrane in hydrochloric acid at temperatures higher than 70 °C permanently reduces its conductivity by a factor of five. Even though no explanation was given for this phenomenon, the reduction in conductivity can be associated with membrane poisoning or degradation, as has been shown by Fox and Colón-Mercado [18], where the Ohmic drop through the membrane drastically increased with ionic impurities. 

In addition to ionic conductivity, the other important property of the Nafion membrane is its ion exclusion ability and separation efficiency. Ideally, Nafion should allow for the transport of only cations (e.g., protons). Proton transport is also associated with water transport due to solvation effects, which is also called osmotic drag. It has also been reported that even other neutral molecules (like methanol, but also gases, like O_2_, chlorine *etc.*) can permeate the membrane. Even HCl crossover might start at higher acid concentrations, as has been discussed above. This was also experimentally confirmed in radiotracer experiments [19]. The authors determined the average HCl diffusion coefficient through Nafion (Table 1) and reported its dependence on membrane pretreatment. Nafion membranes are commonly prepared according to the method of Zawodzinski [14], where they are first boiled in 5% H_2_O_2_, then in 0.5 M H_2_SO_4_ and, finally, rinsed in water. Following this procedure, fully hydrated Nafion 117 membranes showed values of 1.2 × 10^−11^ m^2^ s^−1^. After skipping the boiling steps and only submerging the membranes in water for 24 h and 3 months, values of 1.5 and 3.8 × 10^−12 ^m^2^ s^−1^ were determined, respectively. These results imply that structural changes might occur in Nafion due to it being boiled in an oxidizing media, which could also be extended to electrolysis cycles at high concentrations (industrial acid concentrations of around 18–22 wt % or 5.5–6.5 M are employed) and high temperatures. Representative values for the diffusion coefficients of the relevant species are presented in Table 1.

As we have commented, Nafion separation efficiency decreases with increasing acid concentration. Another important process parameter, which can influence the separation efficiency, is the operating current density. Van der Stegen *et al.* [20] observed that diffusivities through the membrane were affected by the current density and the water content, observing higher permeation rates at higher current densities and higher water contents. DuPont [21] also reported a greater mass flux through the membrane at higher current densities, which, in turn, increased the internal pressure in the polymeric structure and caused membrane swelling, pore enlargement, and an increase of the effective diffusion coefficients. 

**Table 1:** Transport parameters of species involved in chlorine recycling.

Species	Diffusion Coefficient/m^2^ s^−1^	
**HCl**	1.5 × 10^−12^–1.2 × 10^−11^	[15,19]
**H_2_O**	4.9 × 10^−10^–3.5 × 10^−9^	[10,20,22]
**Cl_2_**	1.0 × 10^−11^–1.2 × 10^−10^	[15,21,23]

In addition to the above-described properties, the mechanical and chemical stability of the membrane is important for its industrial application. Nafion membranes should be tolerant to pressure changes, which can occur during operation. For instance, the gas production rate (resulting from the O_2_ evolution due to a side reaction) at low local reactant concentrations can increase, leading to a rise in the cell voltage and gas pressure in the membrane. Both can accelerate membrane degradation. For HCl gas phase oxidation (Equation 1), insufficient membrane humidification might be a problem due to the drying of the CL as a result of the gas flow. The flow-field in such a reactor must be carefully designed and analyzed in order to control the current density distribution along the membrane and the places where gas flow could carry more water away. Non-homogeneous current density distributions can lead to hot-spots, as well as non-uniform membrane swelling, which, in turn, can create a pressure profile along the membrane and leave it prone to fissures or membrane thinning [24]. Membrane thinning is also present during the startup and shutdown of electrolyzers. For instance, the water flux through the membrane can quintuple its value when the current density goes from 500 to 0 mA cm^−2^,causing severe stress in the membrane if the load change is performed in a fast manner [10,21]. This effect is more notorious at higher temperatures. Also membrane poisoning with metal ions can be observed at shutdowns if chlorine is not completely evacuated from the anode compartment before current interruption. The remaining chlorine will be reduced to chloride ions while the metal impurities or the catalyst in the cathode can dissolve and be deposited inside the membrane [21], permanently reducing membrane conductivity and the cathodic active surface area.

### 2.2. Nafion in the Catalyst Layer

The protons produced by electrochemical reactions (e.g., Equations 1-2 and 2-2) must be transported from the anode to the cathode in order to close the electrical circuit. For this reason, a catalyst layer (CL) has to be in contact with an ion-conducting medium. If the reaction is taking place at the dimensionally stable anode (e.g., Equation 2-2), the liquid electrolyte provides the medium for proton transport. On the other hand, for reactions taking place in the CL of the gas diffusion electrodes (Equations 1-1, 1-2, 2-1), the CL has to be impregnated with an additional ion-conducting phase. In the case of low temperature processes, Nafion is the material of choice due to its super acidic and hydration properties, mechanical strength, high conductivity, and inert chemical nature. Ideally, Nafion provides an ion-conducting network in the CL without influencing the properties of the catalyst (noble metal or carbon catalyst), mass transfer and electrical resistances. The question is how far is Nafion from these ideal requirements?

In order to form an ion-conducting network in practice, a Nafion “solution” (typically 5 wt % Nafion in water and/or alcohol) is mixed with the catalyst powder to form a catalyst ink. A self-organization of the Nafion and the catalyst particles already starts at this stage. Nafion strongly adsorbs onto both the carbon and noble metal particles (*i.e.*, Pt), as has been evidenced by nuclear magnetic resonance spectroscopy (NMR) [25]. Interestingly, the Nafion affinity to platinum is lower than to carbon, which implies that the acidic groups on the carbon might participate in the adsorption process. Even different types of carbon show different equilibrium constants, which, in general, increase with an increase in the total BET surface area. In all cases, Nafion adsorption at lower concentrations follows a Langmuir adsorption isotherm (ascribed to primary adsorption), while an additional adsorption isotherm has been observed at higher concentrations (secondary adsorption). It was assumed that the secondary adsorption is related to the presence of nanopores, which correlated well to higher adsorption equilibrium constants in the case of higher surface area carbon nanomaterials with higher inner porosity. 

For a better understanding of the Nafion adsorption on the Pt/carbon catalyst and its relevance for the formation of an ion-conducting network in the catalyst layer, it would be useful to be able to visualize the adsorbed polymer on the Pt/carbon catalyst at a molecular level. At present, this cannot be achieved, but recently, the Nafion behavior on highly ordered pyrolitic graphite (HOPG) (which might be considered as a model surface for Nafion adsorption on carbon) was studied by *in situ* atomic force microscopy (AFM) [26,27]. Koestner *et al.* [26] studied the Nafion adsorption on HOPG from ethanol/water (1:1) solvent at different pHe values on HOPG. They assumed an electrostatic mechanism for the Nafion adsorption, which was further supported by the complete absence of the adsorption at pHe values higher than 4. For pHe values up to 3, Nafion adsorption on HOPG resulted in the formation of segmented rod-like aggregates with a length of *ca.* 100 nm. The pHe value further influenced the thickness of Nafion aggregates, e.g., the thickness was up to 9 nm at a pHe of 1, while it was only 1 nm at a pHe of 3. This result might be important for GDE development, since the thickness of the Nafion layer might influence the transport properties *i.e.*, the mass transfer resistance in the CL. A further interesting result from this study was a very weak Nafion adsorption from the pure ethanol as a solvent, which, in accordance to the authors, was controlled by van der Waals and not electrostatic forces due to the screening of the electrostatic interactions by the low dielectric constant of the solvent. Masuda *et al.* [27] studied the Nafion adsorption from diluted Nafion in water (*ca.* 100 ppm) on HOPG and graphite surfaces. They reported the formation of a worm-like structure after prolonged adsorption on the HOPG in the presence of Nafion in the solution, with heights below 1–2 nm. If the Nafion solution was washed away after 1h of adsorption, aligned ribbon-like wires with a height of 0.3 nm and a width of 5 nm were observed. They also reported that Nafion firstly adsorbs randomly on the HOPG, and then the adsorbed aggregates extend under the influence of the HOPG atomic arrangement. The formation of well-ordered domains was not observed on such surfaces of a low surface symmetry such as glassy carbon. The AFM studies described above were performed under “idealized” conditions, but they still might provide some interesting information relevant for GDE optimization. An interesting observation in both AFM studies was the formation of segmented structures of the Nafion on the investigated surfaces, which might influence the widely accepted representation of Nafion as a thin film uniformly covering the surface. We hope that these very interesting studies will be followed by further investigations under conditions, which are more “realistic”, resulting in a deeper understanding of Nafion/Pt/Carbon interactions. 

The self-organization of Nafion and carbon particles may lead to further formation of agglomerates. The mechanism of their formation is still not clear, but it is known that the level of agglomeration, the particle size and particle size distribution, as well as the porosity and ionic conductivity, depend to a great extent on the dispersion media. As a dispersion media, water-alcohol mixtures but also different organic solvents have been used [28,29]. The property of the dispersion medium, which seems to influence (control) the agglomeration process, is its solubility parameter. Nafion itself has two solubility parameters; the first one corresponds to the perfluorocarbon backbone ([9.7 cal cm^−3^]^1/2^) and the second one to the sulfonated vinyl ether side chain ([17.3 cal cm^−3^]^1/2^) [30]. If the solubility parameter of the dispersion medium is close to that of the Nafion perfluorocarbon backbone, a better dispersion of the Nafion, *i.e.*, a lower level of agglomeration, should be expected. For example, in water (solubility parameter [23.4 cal cm^−3^]^1/2^) Nafion forms two types of agglomerates [31]. Intrinsic (primary) agglomerates are formed due to the hydrophobic interactions of the perfluorocarbon backbone with water, which forces the Nafion chains to associate with each other. This results in the formation of rod like aggregates with hydrodynamic radii from 100 to 200 nm. Intrinsic aggregates can further interconnect through hydrophilic side chain interactions, forming much larger secondary aggregates with radii from 500 to 800 nm. The primary agglomerates can be dissociated using a solvent with a solubility parameter close to that of the Nafion backbone, while the secondary agglomerates can be dissociated by adding some salt e.g., NaCl (0.1 M) into the Nafion solution to shield the SO_3_H ionic interactions. According to the literature, a high degree of chain aggregation and large scale hydrophilic and hydrophobic phase separation are important features to achieve a high ionic conductivity and a high degree of swelling, which has also been experimentally validated in water swelling and conductivity measurements [32]. Nafion membranes casted from *N,N'*-dimethyl formamide (DMF) (solubility parameter 12.2 (cal cm^−3^)^1/2^) or water/alcohol mixtures (solubility parameters from 16.3 to 14.4 (cal cm^−3^)^1/2^) showed different swelling behaviors and different ionic conductivities with the membranes obtained by casting from water/alcohol mixtures taking the lead (water content in final membranes was 35 vol % compared to 12 vol %, and the conductivity was *ca.* 2.7 × 10^−2^ S cm^−1^ compared to *ca.* 1.2 × 10^−2^ S cm^−1^ for water/alcohol and DMF membranes, respectively). 

These interesting fundamental observations have importance for the development of catalyst ink recipes, which has recently been demonstrated by Xie *et al.* [29]. In their study, the performances of gas diffusion electrodes prepared by using water (97 wt %) and alcohol (77 wt % *n*-propanol, 22 wt % water) based catalyst inks were compared in terms of their porosity, electrochemically active surface area (ESA) and oxygen activity. Interestingly, both types of electrodes exhibited similar ESA and similar activity towards the oxygen reaction. The major difference was observed in terms of porosity and proton conductivity. “Water”-based electrodes showed higher porosity (lower oxygen mass transfer resistance), but also lower proton conductivity. The authors could offer only tentative explanations for the observed phenomena, stating that alcohol-based inks were more compatible (in terms of their solubility parameters) with Nafion. Alternatively, the reason for lower ionic conductivity under operating conditions could have been the higher porosity of the Nafion observed in the case of the “water”-based electrodes. It decreased mass transfer resistance on the one hand, but could contribute to a higher level of drying out the CL on the other hand. 

**Figure 5:**
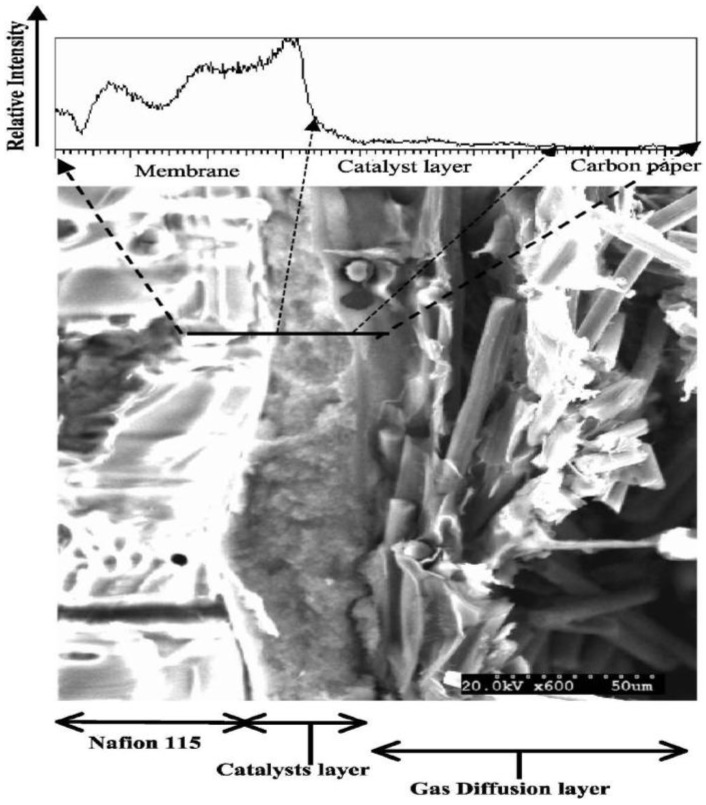
Scanning electron microscopy (SEM) micrograph of a cross-section of the membrane electrode assembly, along with a profile of elemental fluorine across the layers. Reprinted with permission from [33]. Copyright 2012, The Electrochemical Society.

**Figure 6:**
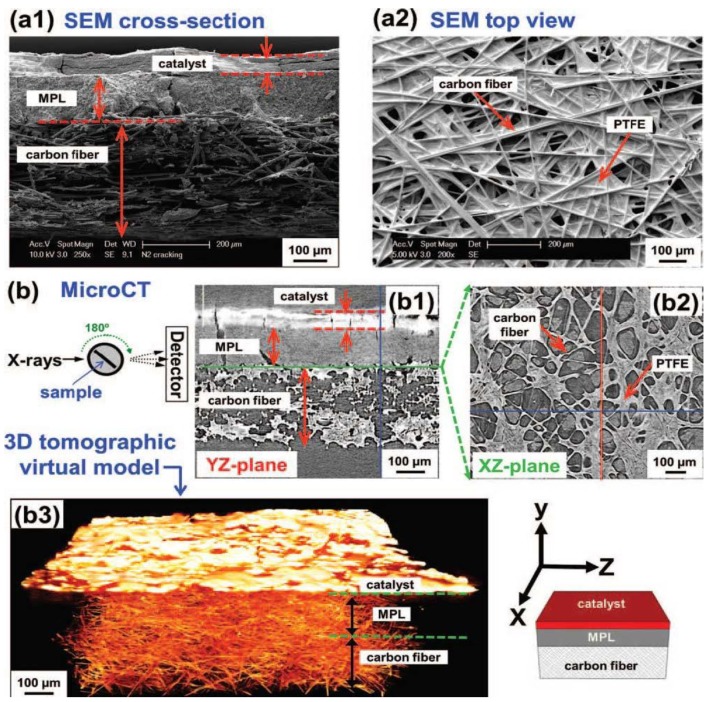
Comparison of the information obtained by traditional SEM cross-section analysis and micro-computed tomography. Reprinted with permission from [34]. Copyright 2012, The Electrochemical Society.

To clarify the observed effects, it would be helpful to assess the properties of the Nafion network in the CL directly. Traditionally, scanning electron microscopy (SEM) can be applied to study GDE electrodes (top or cross section). In this way, the distribution of the Nafion throughout the catalyst layer can possibly be visualized (Figure 5) [33]. Still, this method is destructive and cannot provide *in situ* (under operating conditions) information. Recently, X-ray tomography (Figure 6 [34]) has emerged as a new analytical technique to enable the non-destructive analysis of GDE architecture, liquid water transport, *etc.* [34,35,36]. First results are encouraging, and we hope that further applications of this method, for the detailed understanding of the relationship between preparation conditions and CL performance will follow soon.

**Figure 7:**
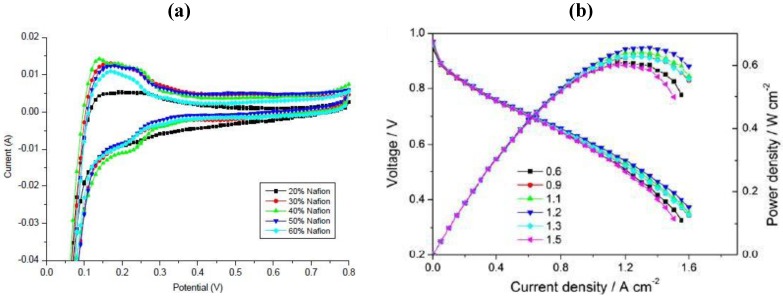
Influence of Nafion loading on (**a**) electrochemically active surface area (ESA) in the presence of nitrogen at 25 °C and 20 mV s^−1 ^and (**b**) fuel cell performance at 65 °C employing GDEs comprising carbon supported platinum catalyst. Reprinted with permission from [37,38]. Copyright 2012, Elsevier.

So far, the best non-destructive assessment of integral electrode performance can be obtained by the application of electrochemical methods in the absence or presence of reactants (e.g., oxygen or HCl) (Figure 7) [37,38]. In the first case, an electrochemically active surface area can be determined. This method is limited to noble metal catalysts, which show a hydrogen under-potential adsorption/desorption region. An example of this kind of catalyst is platinum, which might be used for oxygen reduction or HCl oxidation. Basically, cyclic voltammetry in the absence of reactants (e.g., in a nitrogen atmosphere) is performed and the electrochemically active surface area is calculated from the charge in the hydrogen under-potential adsorption/desorption region assuming a constant charge for a hydrogen monolayer (0.210 mC cm^−2^) [39]. In this way, the ESA of electrodes under technical conditions in an electrochemical reactor or in an electrochemical half-cell can be determined. Lee *et al.* [40] varied the Nafion to carbon ratio in the catalyst layer and found that the Pt utilization increased with an increase in Nafion loading, leveling out at Nafion to carbon ratios higher than 0.75. Similar information was obtained in other studies using gas diffusion electrodes. These studies were further supported by performance tests (polarization curve measurements) in the presence of reactants (e.g., oxygen), which usually led to the assumption that the addition of Nafion increased the so-called three-phase boundary, where it was assumed that the reaction took place under technical conditions. 

Unlike these studies on GDE electrodes, half-cell studies, which utilize catalyst layer intermixed with Nafion (denoted as mixed) or Nafion in form of a thin film on top of the catalyst layer (denoted as coated) reported a different dependence on the Nafion loading. For example, Ignaszak *et al.* [41] studied the influence of the Nafion loading in the range from 0.09 to 1.1 mg cm^−2^ Nafion at a platinum loading of 0.1 mg cm^−2^ in a half-cell using mixed and coated electrodes and perchloric acid as an electrolyte. A decrease in the ESA was observed in both cases, but it was less pronounced in the case of coated electrodes. Similar results were obtained by Passalacqua *et al.* [42], who also observed that the ESA decreased with Nafion loading in the whole investigated range. Ohma *et al.* [43] also found a decrease in the ORR activity on a bulk platinum electrode in the presence of a Nafion film, followed by a higher rate of peroxide formation. They assumed that the oxygen adsorption on the platinum surface was partly blocked by adsorbed Nafion.

Most half-cell studies observe a decrease in the ESA with an increase of the Nafion loading, while tests with GDE under reactor conditions usually show a certain optimal Nafion loading. One can assume that “all” catalyst particles will be in contact with the liquid proton-conducting phase in a half-cell study. The decrease in ESA with an increase of Nafion loading can indicate a higher degree of catalyst site blockage at higher Nafion loadings and/or an increase of the mass transfer resistance. This would further imply an intrinsic decrease in the catalyst activity in the presence of Nafion. As opposed to half-cell studies, GDE under technical conditions show an initial increase of activity until an optimal Nafion loading is achieved, which is then followed by a decrease in activity. It is usually assumed that Nafion provides ionic pathways under technical conditions, which justify an increase in the activity with a Nafion loading in terms of the well-accepted formation of a three-phase boundary. A decrease at higher loadings is usually ascribed to an increase in the mass transfer resistance within the catalyst layer. However, if Nafion blocks the platinum surface in the presence of low anion adsorbing liquid electrolytes (e.g., HClO_4_), it might be natural to expect a similar behavior under technical conditions. To explain this discrepancy in relation to the results obtained in the half-cell studies, it would be useful to revisit the role of Nafion as a primary ion-conducting medium under technical conditions. 

The consideration of Nafion as an ion-conduction medium is based on Nafion’s super acidity, which is supported by the high Nafion proton concentration (3.2 M) [16]. Still, to enable ionic conductivity, these protons have to be in a dissociated form, which requires the presence of water. As already discussed in Section 2.1, only fully humidified membranes can provide sufficient ionic conductivity for the technical operation. We might further assume that catalyst particles will be in contact with liquid water under these conditions, where the electrochemical reactions will also take place. Recently, Eikerling *et al.* [44] revisited the role of water in a fuel cell, stating that only catalyst particles in contact with water will be active for electrochemical transformations. Xie *et al.* [30] have also shown that the ESA was dependent on the relative humidity (RH) changing from *ca.* 58 (100% RH) to *ca.* 40 (30% RH) m^2^ g^−1^. It has also been recently shown that ultra-thin catalyst layers have almost the same activity at reduced metal loadings as state-of-the-art catalyst layers in the absence of Nafion and while using only water as an ion carrier [45]. We might speculate further that water forms an essential ionic-conduction network in the GDE under technical conditions. In this case, the primary role of Nafion will be based on its good hydration properties (high degree of swelling in the presence of water due to the presence of hydrophilic sulfonic groups), which will keep water (primary ion-conducting phase) in place (Nafion protects the drying of the catalyst layer to some extent). 

The presence of Nafion in the CL can also increase the mass transfer resistance of reactants and products of the reaction. Ignaszak *et al.* [41] showed that Nafion top-coated electrodes exhibited lower activity for the ORR reaction, which was ascribed to an increase in the mass transfer resistance for the oxygen transport. The increase in the mass transfer resistance through the Nafion film is usually seen as a main reason for decreases in the GDE activity under technical conditions and at high Nafion loadings. Nafion is not completely impermeable to gases, which allows for the transport of gases in the presence of high driving forces such as concentration gradients. According to data from different authors [14,46,47,48], Nafion can absorb water up to 30 vol %, allowing for gas pathways to be present at low humidification levels. In the presence of high amounts of water or any other polar liquid, continuous liquid channels through the Nafion structure are formed, which are probably more relevant for practical applications. Still, reactants can dissolve in Nafion or water [49,50,51]. In this case and for GDE electrodes under technical conditions, one still profits from shorter mass transfer pathways and a higher driving force for the gas diffusion than is the case in half-cell studies. If the Nafion loading in the CL is very high, it may even break the electron-conducting network, rendering some catalysts inactive. 

Based on the discussion above, it can be assumed that Nafion influences the catalytic properties of platinum catalysts, at least in regards to the ORR. In the case of the HCl oxidation, there are not enough studies in the literature to draw a final conclusion about the effect of Nafion. In addition, Nafion might also increase the mass transfer and electrical resistance in the catalyst layer. The currently low level of catalyst utilization under technical conditions (around 10% according to Xia *et al.* [52]) suggests further improvements, where fine-tuning of the polymer properties could possibly result in a technological breakthrough. In light of the previous discussion, improved polymer materials for low temperature processes should behave as a non-anion adsorbing electrolyte, should possess excellent hydration properties (water is still a green solvent) and have a high level of dissociated protons. 

## 3. Conclusions

We have reviewed chlorine recycling in electrochemical membrane reactors utilizing a Nafion membrane and GDE. An important property of Nafion for its technical application is its proton conductivity. According to the literature, the conductivity of Nafion equilibrated with acid is lower than the conductivity of water-equilibrated Nafion, and it decreases steeply at higher acid concentrations. The decrease of the conductivity might be further followed by an increase in the Cl^−^ crossover. Keeping in mind that the concentrations of hydrochloric acid in technical processes are 18 to 20 wt %, it can be anticipated that both effects might influence the technical processes for chlorine recycling due to the increase in the energy demand and the deterioration of the catalytic activity. Furthermore, Nafion conductivity, separation efficiency, and its chemical and mechanical stability are influenced by membrane pre-treatment (thermal or electro-chemical) and operating conditions. In addition to the properties of Nafion as a membrane, we discussed its role as an ion-conducting medium in the catalyst layer. The problems in the development of the GDE for electrochemical membrane separation processes overlap to a great extent with the development of the GDE for energy applications. In general, the state-of-the-art GDE exhibit low catalyst utilization, which suggests the necessity of further improvements. For a major breakthrough in this area, joint fundamental and applied studies, starting with the influence of the dispersion medium on the properties of the catalyst ink and the formation of the ion-conducting network in the catalyst layer (including its *in situ* visualization), are necessary. This will hopefully lead to a better understanding of the structure-performance relationship. We also pointed out the importance of new experimental methods (like micro-computed tomography) for improved catalyst layer characterization. The role of Nafion as a primary ion-conducting medium and as a non-adsorbing electrolyte was also revisited, giving some evidence that water is an essential ion-conducting medium at low temperature operating conditions. 

## Nomenclature

CL: Catalyst layer
DSE: Dimensionally stable electrode
FF: Flow field
GDE: Gas-diffusion electrode
GDL: Gas-diffusion layer
MEM: Membrane
OCC: Oxygen consuming cathode
ORR: Oxygen reduction reaction
*D_i_^j^*: (m^2^ s^−1^): Diffusion coefficient of species *i* in medium *j*
